# Multi-Target Feeding-Behavior Recognition Method for Cows Based on Improved RefineMask

**DOI:** 10.3390/s24102975

**Published:** 2024-05-08

**Authors:** Xuwen Li, Ronghua Gao, Qifeng Li, Rong Wang, Shanghao Liu, Weiwei Huang, Liuyiyi Yang, Zhenyuan Zhuo

**Affiliations:** 1 College of Computer and Information Engineering, Tianjin Agricultural University, Tianjin 300384, China; 2209028114@stu.tjau.edu.cn (X.L.); hww004@163.com (W.H.); 2 Research Center of Information Technology, Beijing Academy of Agriculture and Forestry Sciences, Beijing 100097, China; rongw@nwafu.edu.cn (R.W.); lshrus@nwafu.edu.cn (S.L.); 202230721124@bua.edu.cn (L.Y.); 202230721135@bua.edu.cn (Z.Z.); 3 College of Information Engineering, Northwest A&F University, Xianyang 712100, China; 4 College of Intelligent Science and Engineering, Beijing University of Agriculture, Beijing 100096, China

**Keywords:** RefineMask, instance segmentation, feeding behavior, behavioral recognition

## Abstract

Within the current process of large-scale dairy-cattle breeding, to address the problems of low recognition-accuracy and significant recognition-error associated with existing visual methods, we propose a method for recognizing the feeding behavior of dairy cows, one based on an improved RefineMask instance-segmentation model, and using high-quality detection and segmentation results to realize the recognition of the feeding behavior of dairy cows. Firstly, the input features are better extracted by incorporating the convolutional block attention module into the residual module of the feature extraction network. Secondly, an efficient channel attention module is incorporated into the neck design to achieve efficient integration of feature extraction while avoiding the surge of parameter volume computation. Subsequently, the GIoU loss function is used to increase the area of the prediction frame to optimize the convergence speed of the loss function, thus improving the regression accuracy. Finally, the logic of using mask information to recognize foraging behavior was designed, and the accurate recognition of foraging behavior was achieved according to the segmentation results of the model. We constructed, trained, and tested a cow dataset consisting of 1000 images from 50 different individual cows at peak feeding times. The method’s effectiveness, robustness, and accuracy were verified by comparing it with example segmentation algorithms such as MSRCNN, Point_Rend, Cascade_Mask, and ConvNet_V2. The experimental results show that the accuracy of the improved RefineMask algorithm in recognizing the bounding box and accurately determining the segmentation mask is 98.3%, which is higher than that of the benchmark model by 0.7 percentage points; for this, the model parameter count size was 49.96 M, which meets the practical needs of local deployment. In addition, the technologies under study performed well in a variety of scenarios and adapted to various light environments; this research can provide technical support for the analysis of the relationship between cow feeding behavior and feed intake during peak feeding periods.

## 1. Introduction

Feeding behavior is one of the primary behaviors affecting dairy cows’ growth, development, and lactation performance [1,2]. Feeding time is an essential indicator of an individual cow’s health status and a necessary basis for evaluating feed utilization and feeding efficiency, as well as a adjusting the decision-making processes of dairy farms [3,4,5,6]. Kazemi et al. investigated a model for the relationship between cow feeding-behavior and cow yield, and the experimental results showed that cow feeding time was positively correlated with cow milk production [7]. Martins et al. outlined the relationship of inflammation to metabolism and nutrition in dairy cows, noting that metabolic disorders are closely related to cow feeding-behavior [8]. Therefore, effective monitoring of cow feeding-behavior can provide a basis for evaluating cow health, which positively impacts the sustainability and economic efficiency of dairy farming [9,10]. Traditional monitoring of the feeding behavior of dairy cows mainly relies on manual tracking, which is inefficient and costly, and cannot guarantee the accuracy of this method [11].

With the continuous development of modern animal husbandry, precision farming with modernized farming operation technology and management has gradually received the attention of researchers, and the recognition of the accuracy of the feeding behavior of dairy cows has become a bottleneck, restricting precision feeding [12,13,14]. Large-scale cattle farms rely on traditional methods to obtain only a rough idea of how the herd is being fed. We need intelligent monitoring methods to identify cow behavior for fine-grained feeding [15,16,17,18]. The three main types of contact sensors include sound sensing, pressure sensing, and acceleration sensing [19]. Stygar et al. investigated collars with pressure sensors to monitor dairy cows’ ruminating times, feeding times, and resting times. The pressure sensor collars were embedded with an intelligent algorithm which used the frequency of temporal fossa vibration during feeding to recognize feeding behavior with high recognition accuracy [20]. Navon et al. collected sound data generated by the movement of cows’ upper and lower jaws through sound sensors and used an algorithm to remove noise for sound classification [21]. In addition, Zambelis et al. identified feeding behavior, using acceleration ear tags, with over 95% accuracy [22]. The accuracy of contact sensors meets the criteria for identifying the feeding behavior of cows. Still, research exists that suggests that contact devices have some limitations, such as the potential for skin abrasions and device-loss issues with wearable devices [23]. Stephanie Buijs et al. evaluated the effects of head- and neck-mounted wearables on cows by designing a 2×3-week experiment and found that the milk yield and milk lactose content of cows were significantly reduced [24]. Wearables that pose a risk to welfare or productivity are therefore unlikely to be widely used.

Within today’s wide application of new-generation artificial intelligence technology, the vision-based dairy cow feeding-behavior recognition method can avoid negative impacts on dairy cow production and save the cost of human observation, a fact which is expected to realize large-scale promotion [25,26]. Porto investigated target detection algorithms used to identify behaviors such as lying down, head milking, eating, drinking, and standing in dairy cows, and the results showed that the recognition accuracy levels for drinking and eating were low. It can be seen that it is difficult for the target detection model to achieve better recognition of interactive behaviors such as drinking and eating [27]. Yu Zhenwei et al. improved the model’s deep learning and feature extraction enhancement. They enriched the scale semantic feature interactions by replacing the CSPDarknet backbone with the independently designed DRNet backbone using multi-feature scales and a spatial pyramid aggregation (SPP) structure based on the YOLOv4 algorithm [28]. Bai Qiang et al. proposed an improved multi-scale behavior recognition method for dairy cows in YOLOV5s. In this study, a new idea was suggested, that of achieving the recognition of cow feeding-behavior based on the target detection results for the cow’s head and the feed pile, which achieves an improvement of the recognition effect as to the cow’s feeding behavior and breaks through the bottleneck of the low accuracy of the existing target detection methods for the recognition of interactive behaviors [29]. In the above study, it isn’t easy to ensure the accuracy of direct identification of the feeding behavior of cows, and the error in calculating feeding time is extremely high. There is also room for improvement in the recognition method based on the target detection results because the arching action of cows during the feeding process leads to highly irregular feed accumulations [30,31]. The location information generated by the target detection method is not sufficiently fine to accurately identify the feeding behavior of cows, so it is necessary to rely on more precise instance segmentation methods to determine the location information [32]. Bello et al. achieved average recognition accuracies of up to 93.34%, 88.03%, and 93.51% for dairy cows’ eating, activity-based, and resting behaviors, using the MaskRCNN segmentation method. Still, the quality of the masks for intensive feeding scenarios needs to be improved [33]. The RefineMask algorithm predicts mask information more accurately, compared to classical segmentation methods, and the segmentation detector head added on top of the original detector is more suitable for the segmentation task of predicting boundaries, although underutilization of global feature information is caused by its enhanced boundary perception [34]. Large-scale cattle farms adopt centralized spreading, which causes interference associated with other heads during the peak feeding period and produces high background noise in the feeding area, so it is necessary to improve the model’s sensory field and feature extraction capability to achieve accurate segmentation of heads and piles.

Aiming at the problem of the low accuracy of existing vision methods as to recognizing the feeding behavior of cows in group feeding environments, this study develops a process that combines the attention mechanism with backbone networks and feature pyramids. The best base segmentation model is selected by using the cow feeding-behavior dataset in the group feeding scenario. The feature extraction, feature fusion, and loss function parts of the base model are optimized to improve the accurate segmentation of the cow’s head and the feed pile, and ultimately to achieve the high-precision recognition of the cow’s feeding behavior.

## 2. Materials and Methods

### 2.1. Data Sources

**Figure 1:**
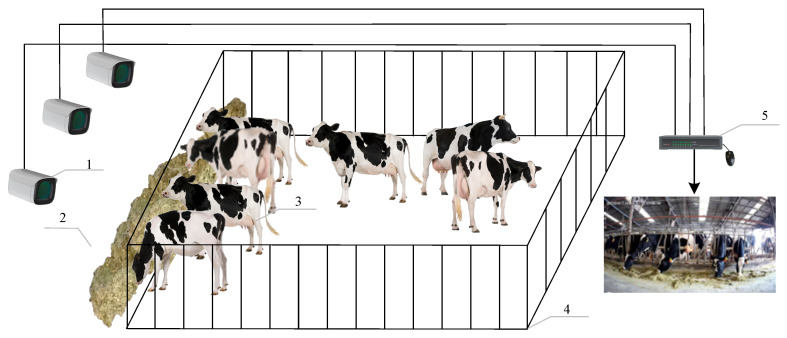
Environmental map of dairy-cattle-feeding data collection: (1) HD camera; (2) Feed trough; (3) Cow; (4) Fence; and (5) NVR.

From February 2023 to September 2023, video data were collected from the peak feeding periods of dairy cows at the Yanqing Dadi Qunsheng Dairy Farming Base in Beijing, China. The study’s scenario was limited to a real farming scene, and the filming time was limited to the end of spreading, when the cows were at their peak feeding period. To ensure the generalizability of the algorithm to real farming scenarios, the dataset has the following properties: (1) Postural variability, which includes head-on and head-down views of foraging behavior to ensure the diversity of the sample data. (2) Light variability, which describes feeding behaviors under different light conditions, such as daytime with light, daytime without light, and night, to avoid the interference of light in the model’s performance. (3) Contextual variability, the inclusion of in-house and out-of-house feeding behaviors, enhances a model’s adaptability to variable environments. Under the above conditions, a full-color cartridge network camera (DS-2CD3T87WDV3-L, Hikvision, Hangzhou, China) with a focal length of 6 mm, 8 megapixels, a resolution of 3840 pixels × 2160 pixels, and a frame rate of 20 fps was used as the leading collection device. A distortion-free handheld digital video camera (FDR-AX45A, Sony, Tokyo, Japan) with a focal length of 35 mm and 8 megapixels was used as a supplementary acquisition device to capture video data on the feeding behavior of cows with a resolution of 3840 pixels × 2160 pixels and a frame rate of 25 fps. In summary, in accord with the filming requirements, it was proposed to set up HD cameras in different feeding environments, with lenses ranging from 1.5 m to 2.5 m from the fronts of the feeding cows, filming two viewpoints, front view (at a height of 1.2 to 1.6 meters) and elevation view (at a height of 0.4 to 0.6 meters), and continuously capturing video data during multiple periods during the daytime and nighttime; the captured images were all RGB-colored images, and the captured video was stored on a hard disk recorder (NVR, the HIKVISIONDS-8832N-K8, Hikvision, Hangzhou, China), as shown in Figure 1.

**Figure 2:**
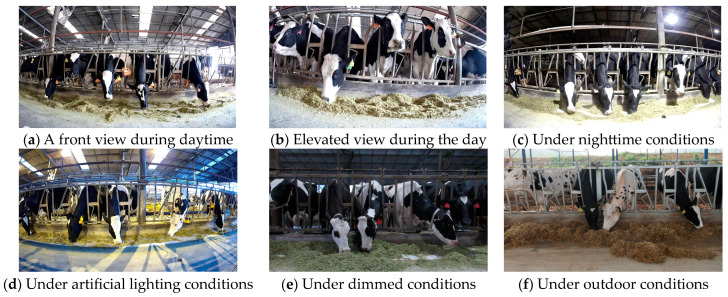
Cow feeding-behavior data.

Data on cow feeding-behavior are shown explicitly in Figure 2, demonstrating examples of cow feeding-behavior under different angles, times, and backgrounds. Figure 2a,b show cow feeding-behavior under different angular viewpoints during the daytime, Figure 2c shows cow feeding-behavior during the nighttime, Figure 2d,e reflect the differences in lighting in indoor environments, and Figure 2f shows the outdoor cow feeding area.

### 2.2. Dataset Production

To improve the model’s generalization ability and reduce the similarity of the images in the dataset, the acquired video data of cow feeding-behavior is first pre-processed, and the cow feeding video is converted into image data by inter-frame interception. In order to investigate the optimal solution for video frame-by-frame interception, videos in multiple scenes are converted into images frame-by-frame and composed into image pairs according to different interval frames; a total of 3000 image pairs are obtained. The MSE (mean squared error) algorithm (1) and PSNR (peak signal-to-noise ratio) algorithm were used to calculate Equation (2), in order to characterize the similarity of the two images and plot the correlation curves. The larger the MSE value, the larger the mean square error of the two images and the lower the degree of similarity of the pictures. The lower the PSNR, the smaller the peak signal-to-noise ratio and the higher the distortion of the second image compared to that of the first image.

(1) $$MSE=\frac{1}{M\cdot N}\sum_{i=1}^{M}\sum_{j=1}^{N}{\left(I(i,j)-K(i,j)\right)}^{2}$$

(2) $$PSNR=10\cdot \log_{10}(\frac{MAX_{I}^{2}}{MSE})$$

In Equation (1), where M and N denote the length and width of the image, respectively; *I(i, j)* and *K(i, j)* denote the component grey values corresponding to the red, blue, and green color channels in the pixels where the corresponding points are located in the two images, respectively; and in Equation (2), where $MAX_{I}$ represents the maximum number pixels in the image with grey values.

**Figure 3:**
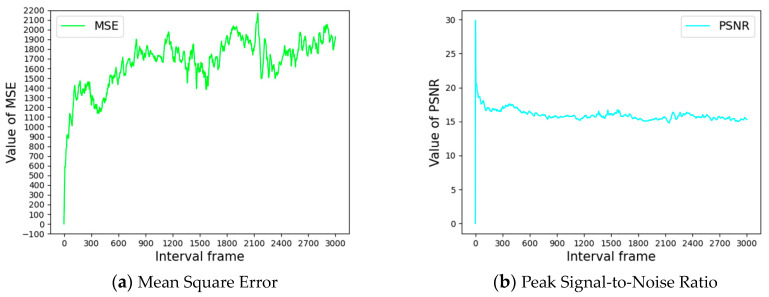
Interval frames with corresponding MSE values and PSNR values.

As can be seen from Figure 3, when the frame interval reaches 600 frames, the MSE dot-plot no longer surges, and the PSNR curve maintains fluctuations within a specific range, so it was finally determined to remove image redundancy by drawing a frame every 600 frames. After removing the redundant images, the training set, validation set, and test set of the cow feeding-behavior data were divided according to 8:1:1, and 800 training sets, 100 validation sets, and 100 test sets were obtained.

Cows feed with their heads outside the restriction fence and come into contact with the feed pile, so labeling information was defined for the feed pile and cow head data. Labeling the dataset according to the definitions in Table 1 resulted in 4291 cow head labels and 1581 feed trough labels.

### 2.3. Data Enhancement

**Figure 4:**
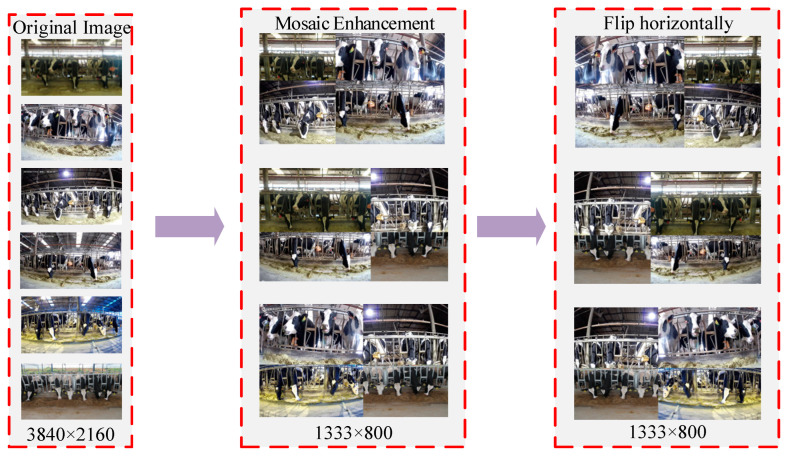
Data enhancement flowchart.

A mosaic approach was employed to enhance the data on cow feeding-behavior to improve model generalization under different light and at different scales. As shown in Figure 4, the process randomly selects and resizes four images from the dataset to obtain an image with dimensions of 800 pixels by 800 pixels. By stitching four images together, an image with dimensions of 1333 pixels by 800 pixels is obtained. Following the affine transformation of the stitched image, mosaic enhancement is achieved by random panning, scaling, cropping, etc.

Finally, the randomly selected images were flipped horizontally to obtain a scale-transformed, more generalized dataset of cow feeding behavior, which was preprocessed as shown in Table 2.

## 3. Recognition of Feeding Behavior in Dairy Cows

### 3.1. RefineMask Network Structure

RefineMask is a model that focuses on improving the boundary segmentation accuracy in the instance segmentation algorithm, and which has high recognition precision and good boundary segmentation effects and meets the requirement for high quality of feed-pile boundary segmentation in the recognition of feeding-behavior of dairy cows, so RefineMask was chosen as the base model. The RefineMask network structure consists of three parts: feature extraction, feature pyramid network, and prediction branch.

The feature extraction part of the model adopts the ResNet50 structure to downsample the input features, explicitly using the Conv_N structure consisting of stacked multiple CBR modules to construct the multilayer residual mapping. In this case, each CBR module avoids gradient vanishing and gradient explosion through the chain rule, which ensures computational efficiency while reducing the use of parameters. Meanwhile, due to the irregular shape of the feed pile, which increases the difficulty of semantic information integration, the feature pyramid network can be used to aggregate semantic information from multiple layers effectively. The semantic information obtained layer by layer from the feature extraction part is convolved, upsampled, summed, and maximally pooled to obtain five feature maps at different scales. To ensure the diversity of fused features, the network structure combines low-resolution, robust semantic features with high-resolution, weaker semantic features. In addition, the cow feeding-behavior recognition method is based on high-quality pixel-level segmentation of the cow head and the cow pile, which requires a high level of perception of the segmentation boundary. Therefore, using a semantic fusion module (SFM) in the prediction branch, which fuses four feature maps containing different semantic information, can improve the network’s ability to predict the details of the mask boundary. This branch utilizes the semantic header and the mask header. It undergoes an upsampling operation to obtain features of larger size, an aspect which is suitable for the boundary prediction of irregular feed piles.

### 3.2. Improvement of the RefineMask Dairy Cow Feeding-Behavior Recognition Model

**Figure 5:**
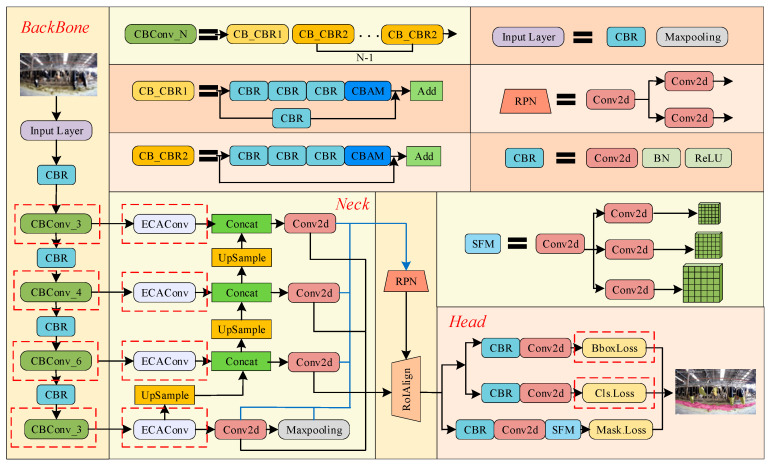
RefineMask−CEG model structure diagram.

The mask prediction ability of the RefineMask base model is relatively excellent. However, in the face of complex scenarios and background noise interference in the feeding environments of dairy cows, the model still suffers from the problems of limited sensing of the backbone network, insufficient integration of features, and inaccurate positioning of the detection box of the model. To ensure the accuracy of the judgment of cow feeding-behavior, this study improved RefineMask by integrating the channel and spatial attention mechanism into the residual mapping structure of ResNet to enhance the feature extraction capability. After that, we designed feature enhancement modules to optimize high-level semantic features in the feature pyramid network. In addition, the loss function and detection frame generation are optimized to speed up model convergence. In Figure 5, the location of the improvements is indicated by a red box. The improved model is named RefineMask-CEG.

When the cow feeding image is fed into the RefineMask-CEG model, the low-level semantic information and high-level semantic information of the image are extracted layer by layer by the improved CB_Conv module, and five feature maps, C1, C2, C3, C4, and C5, at different levels, are finally generated. Then, the feature maps containing different levels of semantic information are fed into the feature pyramid network of the fused ECA_Conv module to achieve different levels of semantic feature aggregation to recognize cow heads and feed piles at different scales. Finally, the aggregated multi-scale feature maps are fed into the prediction branch, and the improved IoU Loss function effectively correlates the four-point coordinates of the model detection frames to obtain the accurate detection frames of the cow head, the feed pile, and the prediction masks.

#### 3.2.1. Feature Extraction Layer Fusion Convolutional Block Attention Module

**Figure 6:**
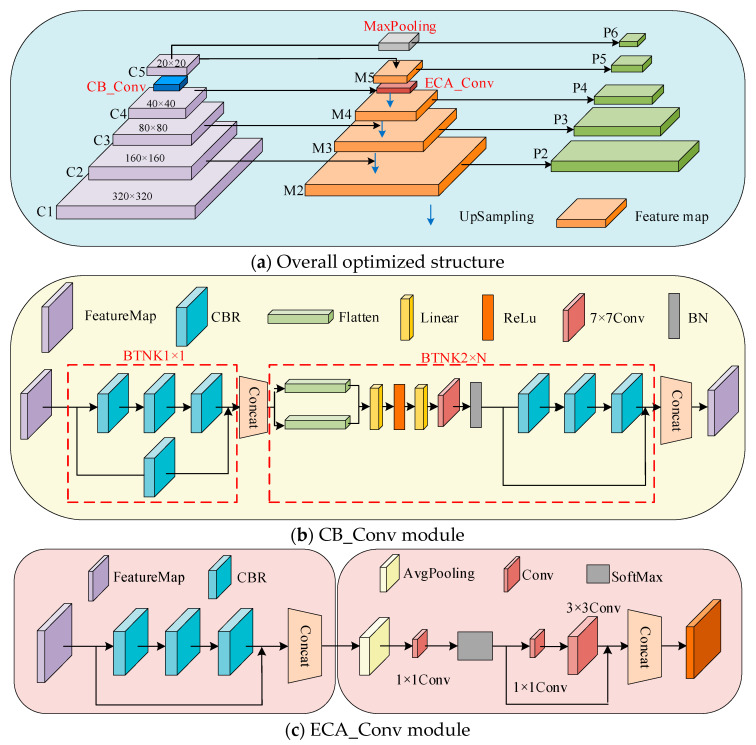
Improved ResNet_FPN network architecture diagram.

The aim of this section of the paper is to address the accurate segmentation of cow heads and feed pile edges in the context of a complex dairy farm environment, which poses significant challenges to the segmentation model. Specifically, the model must have strong feature extraction capability while suppressing cluttered background interference information. This study incorporates channel and spatial attention mechanisms into the feature extraction network structure, constructs the CB_Conv module with strong feature extraction capability, and adaptively learns the attention weights of different semantic layers. The location of the CB_Conv module is shown in Figure 6a, which obtains the global statistical information of each channel by using global average pooling and global maximum pooling and optimizes the multilayered semantic information by expanding the perceptual field of the model, which can effectively solve the problem of insufficient bullhead feature extraction under the influence of background noise. It includes multiple components such as Faltten, Liner, ReLU, CBR, etc. The global and local information associated with cow feeding images can be better learned by using average pooling to obtain aggregated spatial information and maximum pooling to collect essential features.

The overall flow is shown in Figure 6b, where the feature $F\in R^{C*H*W}$ is obtained from the output of BottleNeck1. A one-dimensional convolutional operation is performed on the features. Global average pooling and global maximum pooling are used to obtain global statistical information for each channel, respectively. Then, the resulting feature maps are processed using a shared multilayer perceptron (Shared MLP), and the results are summed up. In this process, the number of channels is compressed to 1/r times the original number of channels and then expanded to the actual number of channels, and weight parameters with coefficients ranging from 0 to 1 are obtained after an activation function. Finally, the weight coefficients are multiplied with the original feature map to change back to the size of C∗H∗W to obtain the feature map Mc(F). This is accomplished by:

(3) Mc(F′)=σ(MLP(AvgPool(F))+MLP(MaxPool(F)))

In Equation (3), F denotes the input feature, and MLP is a multilayer perceptual machine. The σ is the affine transformation in which the pixel points in each layer of the spatial feature are given different weights. Furthermore, the AvgPool is the average pooling, and the MaxPool is the maximum pooling.

Subsequent features will be fed into the pooling layer to complete the spliced feature map, and the feature map will be obtained through a 7 × 7 convolution operation with activation function Ms(F). This is performed by:

(4) $$Ms(F\prime \prime )=\sigma (f^{7\times 7}([AvgPool(F\prime );MaxPool(F\prime )]))$$

In Equation (4), where *F*′ denotes the feature map after channel attention weighting, the $f^{7\times 7}$ represents a convolution operation of size 7 × 7. In addition, the σ refers to the capture-channel-dependent feature transformation.

Finally, the resulting feature map is fed into BottleNeck2 to form a complete CB_Conv module.

#### 3.2.2. Feature Fusion Partially Incorporates an EFFICIENT Channel Attention Convolution Structure

The convolutional-neural-network forward process contains more semantic information and less feature information in deeper networks, while the opposite is true for shallow networks. Therefore, in order to obtain more feature information and semantic information for targets at different scales, improving the feature fusion part is a common means. The main targets recognized by the model in this study include the cow’s head and the feed pile. Due to the smaller scale and higher resolution of the cow head and the larger scale and lower resolution of the fodder pile, the original feature pyramid network structure is unable to effectively characterize the cow head and the fodder pile at different scales simultaneously through a single-layer feature map. The simple fusion of shallow and deep feature information can improve the recognition accuracy for cows’ heads and feed piles, but it will increase the parameter calculation of the network, and it is difficult to ensure that the recognition accuracy of cow head and feed pile is maintained in balance. To address the above problems, in this study, an improved attention mechanism block based on an excitation network (ECANet) and named ECA_Conv block is proposed to capture long-distance dependencies, one which utilizes each channel and its k neighbors to capture local cross-channel interaction information.

The ECA_Conv module consists of CBR, SoftMax, and convolutional layers as shown in Figure 6c; the feature map $F\in R^{C*H*W}$ outputs the convolutional blocks stacked by CBR. After a one-dimensional convolution operation followed by SoftMax excitation, the inputs are fed into a 1 × 1 convolution and a 3 × 3 convolution, respectively, and the results obtained from both are summed up. This module enables global position recalibration by calculating feature positions and the relationship between all positions in an aggregated feature; the formula is in the following form:

(5) $$F_{out}=\delta (\sum_{j=1}^{k}w^{j}y_{i}^{j}),y_{i}^{j}\in \Omega_{i}^{k}$$

Given the channel dimension C, the kernel size k can be determined, as adapted in:

(6) $$k=\psi (C)={\left|\frac{\log_{2}(C)}{r}+\frac{b}{r}\right|}_{odd}$$

In Equation (5), in which k denotes the adaptive determination of kernel size, the values of j are taken from 1 to k, and the values of i are taken from 1 to C. In addition, the $w^{j}$ refers to the learning channel attention parameters, the $y_{i}^{j}$ represents the weight factor, the $\Omega_{i}^{k}$ stands for k neighbors of the $y_{i}^{j}$, and the δ implies an activation function.

In Equation (6), r and b are all parameters of the linear mapping, and the ψ refers to the linear mapping function of k and C.

Since the visualization channel features have a certain local periodicity, only the information exchange between the current channel and its k neighboring channels is considered, with the number of parameters being k×C.

#### 3.2.3. Real Standardized Bounding-Box Regression IOU Loss Function

Bounding-box regression is one of the most fundamental components of computer vision tasks, and tasks such as target detection, target tracking, and instance segmentation rely on accurate bounding-box regression. The original RefineMask model used the L1 loss function, which has the advantage that the gradients are stable regardless of the input values, which averts the gradient explosion problem and is highly robust. The disadvantage is also apparent: the gradients are all equal, which means that even if the loss values are small, their gradients are large, which may not be conducive to the convergence of the function and the learning of the model. Meanwhile, when calculating the regression loss, it is assumed that the four coordinate points are independent of each other, ignoring any correlation between the coordinate points, which leads to the inability of the model to truly reflect the advantages and disadvantages of the detection effect for the cows’ heads and the feed piles. The intersection-over-union calculation gives better feedback on the detection effect by correlating the four-point coordinates with each other while having scale invariance, overcoming the drawbacks of the L1 loss function. Suppose now that there are two arbitrary boxes, *A* and *B*, with *IoU* formulas in the shape of:

(7) $$IoU=\frac{1}{U}=\frac{1}{A^{p}+A^{g}-1}$$

In Equation (7), in which $A^{p}$ denotes the area of box *A*, the $A^{g}$ stands for the area of box *B*. However, there is a problem with the *IoU* loss; when the two objects do not cover each other, the loss function is not derivable, and the *IoU* loss cannot optimize in cases where the two frames are not intersecting. Therefore, this paper uses a more efficient loss function to solve the imbalance problem in the bounding-box regression. Compared to the previous loss function, the *GIoU* loss significantly improves convergence speed and detection accuracy. The principle of *GIoU* is obtained by finding the most minor closed shape *C* which encloses *A* and *B*. The ratio of the calculated area of *C* that does not cover *A* and *B* is compared to the calculated total area of *C* and subtracting this ratio from the *IOU* values of *A* and *B* obtains *GIoU*. The formula is as follows:

(8) $$GIoU=IoU-\frac{A^{c}-U}{A^{c}}$$

(9) $$L_{GIoU}=1-GIoU$$

In Equation (8), $A^{c}$ denotes the area of box *A*. In Equation (9), $L_{GIOU}$ denotes the loss function of *GIoU*.

#### 3.2.4. Evaluation Indicators for Recognition of the Cow Head and the Feed Pile

Qualitative and quantitative aspects were chosen to be evaluated to validate the model’s performance. For qualitative evaluation, the performance of the model is assessed by comparing the difference between the visualized prediction results of RefineMask_CEG and other methods, i.e., comparing the localization accuracy of the target detection frame with the coverage accuracy of the mask prediction results, and determining whether there is any leakage or misdetection. For quantitative evaluation, this paper uses the mean average precision (*mAP*) as the evaluation index used to reflect the training accuracy of the model. The number of parameters, computation, and model weights represent the complexity of the model. The frame rate (FPS) represents the speed of model detection. Specifically, the average precision mean *mAP* is the mean value of average precision (*AP*), and the average precision *AP* is the area of the P-R curve, as shown in Equation (10):

(10) $$mAP=\frac{\sum_{i=1}^{N}\int_{0}^{1}P(R)dR}{N}\times 100\%$$

The N denotes the number of categories, and the topics discussed in this study are the cow head and the feed pile, so in this equation, N=2.

Within which P denotes the ratio of the prediction algorithm area to the actual detection area, the algorithm is shown in Equation (11):

(11) $$P=\frac{T_{p}}{T_{p}+F_{p}}\times 100\%$$

where $T_{P}$ denotes the number of samples correctly predicted as positive, and $F_{P}$ denotes the number of samples incorrectly predicted as positive, R then represents the proportion of correctly predicted samples to all positive samples, as shown in Equation (12):

(12) $$R=\frac{T_{p}}{T_{p}+F_{N}}\times 100\%$$

where $F_{N}$ denotes the number of samples that were incorrectly predicted to be negative and N denotes the number of categories.

#### 3.2.5. Cow Feeding-Behavior Recognition Using Mask Information

**Figure 7:**
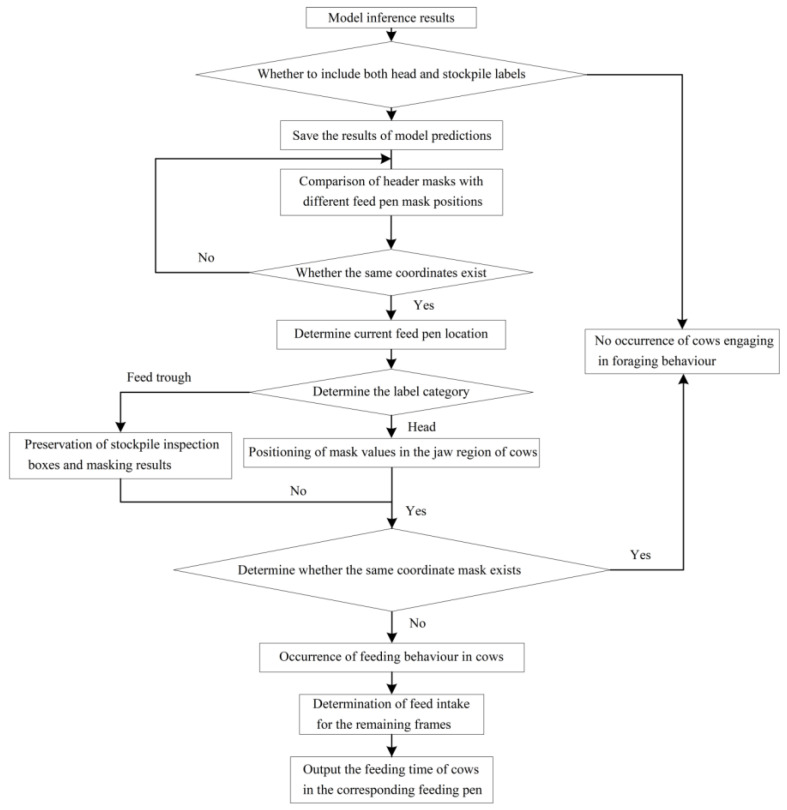
Flowchart of the method for identifying feeding-behavior in dairy cows.

Based on the observation of the data obtained during the peak feeding periods of cows, it is known that hungry cows start to feed at the moment when their head and the feed pile come into contact; this is used to design a method for identifying the feeding-behavior of cows using the mask information as shown in Figure 7. The first step is to input the pictures of cows feeding into the RefineMask-CEG network to evaluate whether the cow head and feed pile labels exist. If these exist, the corresponding coordinate information and mask results are saved. The second step is to obtain the related mask binarization result for the cow’s head and determine whether the head and the feeding fence have exactly the same coordinates, and thus determine the cow’s feeding-fence information. The third step is to obtain the mask results of the jaw portion based on the head mask in the saved cow-head and feeding-fence labels, traverse the feeding fence to obtain the mask results in the same region of the feeding fence, and determine whether the coordinate information is the same between the two, thereby realizing the identification of the feeding-behavior of the cow during the peak feeding period. In the fourth step, the feeding situation of the cows in the rest of the frames is integrated, and the number of feeding frames of the cows under each feeding fence is calculated statistically to calculate the feeding time of the cows.

## 4. Tests and Analysis of Results

### 4.1. Experimental Platform and Model Testing Metrics

This paper’s experiments utilized the Linux operating system, Pytorch deep learning framework, and four 16 GB Tesla P100 GPU servers; the Python version was 3.8, the ubuntu version was 18.04, and the CUDA API version was 10.2. This study uses the same dataset and training strategy to train Mask RCNN, Mask Scoring, Point Rend, and RefineMask-CEG. The mainstream training strategy recognized in the field of instance segmentation uses an image batch size of 16, an image size of 1333 pixels × 800 pixels, a learning rate of 0.02, and 8 GPUs trained in parallel. In this study, to achieve the full utilization of GPU memory storage, the image batches are dynamically adjusted with the learning rate as follows: the image batch size is 8; the learning rate is 0.01 and becomes one-tenth of the prior value after 60,000 and 80,000 iterations; and finally, the training ends after 90,000 iterations. Cow feeding-behavior is judged according to the model’s mask prediction of the cow’s head and the feed pile, and the model’s recognition of both has a direct impact on the recognition of feeding-behavior, so the detection frames and mask recognition of the two labeled categories of cow’s head and feed pile are analyzed. Model performance was assessed using several metrics, such as mean precision, mean average precision, model size, and run time for both categories.

### 4.2. Comparison of Model Identification Results

MaskRCNN, MSRCNN, Point_Rend, Cascade_MaskRCNN, RefineMask, and RefineMask-CEG were trained using the same training set of cow feeding-behaviors. The performance of different example segmentation models were analyzed in terms of mean detection box average precision, mean mask average precision, and model size to evaluate the performance of other example segmentation models, and the results are shown in Table 3.

As can be seen from Table 3, RefineMask-CEG improves the average detection frame mean accuracy by 1.2%, and the average mask means accuracy by 0.8% compared to the classical algorithm MaskRCNN. Compared with MSRCNN, Point_Rend, Cascade_MaskRCNN, and ConvNet_V2, the average precision of detection frames is improved by 0.5%, 1.2%, 1.4%, and 0.6%, and the average precision of masks is improved by 0.9%, 0.7%, 0.9%, and 1%, respectively. Compared with the RefineMask model, the average precision of both detection frames and masks is improved by 0.7%, indicating that the improved model has some degree of improvement in feature extraction and feature processing. Regarding model run speed, the MaskRCNN model runs at 2.3 frames per second. The RefineMask and RefineMask-CEG models were run at 1.4 and 0.8 frames per second, respectively. Model run speeds all decrease somewhat as model accuracy increases. Due to the increase in model calculations with the addition of the new module, there is an unavoidable increase in runtime and a certain degree of slowdown in model runtime.

In terms of model size, the MaskRCNN model size is 44.17 M, and the model sizes of MSRCNN, Point_Rend, Cascade_MaskRCNN, and ConvNet_V2 are 60.51 M, 59.99 M, 77.10 M, and 110.59 M, respectively, with an increase of more than 10 M over MaskRCNN. The RefineMask model size is 48.34 M, an improvement of only 4.17 M over MaskRCNN. The RefineMask-CEG instance-segmentation model proposed in this paper has a size of 49.96 M, and there is no surge in the model size based on RefineMask, which is has a lower profile than the other improved models, while the mAP is the highest among all models. Comprehensively analyzing various data, the RefineMask-CEG model has a significant advantage as to balancing model size and recognition segmentation accuracy.

To further reflect the extraction of target features and the inference prediction ability of the models, the detection box and mask inference results of the six models for the two label categories are compared, as shown in Table 4.

From the above table, we can see that the average frame detection precision and the average mask precision of RefineMask-CEG for the bullhead region are 97.3%, which is 0.9% higher than that of MaskRCNN and 0.8% higher than that of RefineMask. Compared with the newly released ConvNet_V2, they are 0.8% and 1.1% higher, respectively, which indicates that the CB_Conv module in the backbone network can acquire the real features of the full pile in the presence of background noise interference. In addition, since the target size of the full pile is much larger than that of the bullhead, the recognition accuracy for the pile is generally higher than that of the bullhead. Compared with MaskRCNN, RefineMask-CEG achieves an average detection frame and mask precision of 99.3% and 99.2% for feed piles, which is an improvement of 1.4% and 0.5%, respectively. Compared to RefineMask, both are improved by 0.5%. Compared with ConvNet_V2, they are 0.4% and 0.8% higher, respectively. This indicates that the neck ECA_Conv can effectively integrate target features at different scales. As to all the data, RefineMask_CEG has an advantage over MSRCNN, Point_Rend, Cascade_MaskRCNN, and ConvNet_V2, which fully reflects the excellent improvement effect of the improved model.

### 4.3. Ablation Experiments

The RefineMask_CEG model proposed in this paper is based on RefineMask and obtained by adding the feature extraction enhancement module CB_Conv, improving the feature aggregation module ECA_Conv, and optimizing the loss function GIoU Loss. The model performance of RefineMask_CEG was validated using the control-variable method for ablation comparison tests.

To specifically demonstrate the effectiveness of each improvement strategy, as shown in Table 5, experiments were conducted for RefineMask, RefineMask + CB_Conv, and RefineMask + ECA_Conv, respectively. In addition, to analyze the necessity of the improvement for the GIoU Loss function and the experimental effect of the combination of the two improvement strategies, CB_Conv and ECA_Conv, RefineMask + CB + ECA was included in the comparison test. From the ablation comparison test, it can be seen that each improvement strategy improves the detection and segmentation performance of the model to a different extent, and RefineMask + CB_Conv improves the mean value of the average precision of the detection box and mask by 0.5% and 1%, respectively, compared to RefineMask. The collected images of cow feeding-behavior are greatly affected by cow head interference, background noise, and other factors; the use of CB_Conv can expand the model’s sensory field, fully optimize the multi-layer global semantic information, and improve the recognition results for the detection of the box relatively obviously. If too much attention is paid to the global features of the image, the details of the image may be lost. The model must acquire local information on the original image to improve the recognition accuracy of cow heads and feed piles in the case of group farming. RefineMask + ECA improves the mean value of the average accuracy of the detection box and mask by 0.7% and 0.3%, respectively, compared to RefineMask, which partly proves that ECA_Conv can fully capture the local cross-channel interaction information and reduce the feature loss due to the expansion of the receptive field.

To address the problem that the scale difference between the head and the feed pile in the cow feeding image is too significant, the model must be able to extract global features and important features simultaneously and establish the dependency relationships between global features, channel features, and spatial features. There is a large amount of redundant information in the features extracted by the CB_Conv module for each layer, and this needs to be culled by expanding the perceptual region. However, expanding the perceptual region of the model will simultaneously cause a certain degree of feature loss, and a single ECA_Conv module has the problem of feature loss in the feature aggregation stage. In this study, we combine the two to capture the critical information and long-distance channel dependencies between the cow’s head and the feed pile, remove redundant features, and reduce the loss of practical features. The specific effect is reflected in the results of RefineMask + CB + ECA, which show a 0.6% and 0.5% improvement over the average RefineMask detection box and average mask accuracy mean values, respectively. However, compared to RefineMask + ECA, the average precision improvement of the detection box becomes a little worse, although the mask precision is improved. Overall, the RefineMask-CEG model shows slight improvements in detection box accuracy and mask prediction accuracy compared to RefineMask + CB + ECA, which suggests that the GIoU improvement strategy positively impacts the model improvement.

In summary, the various optimizations of RefineMask in this study improved the precision of the model in identifying cow heads and feed piles. Each improvement strategy provided a different contribution to the identification of cow feeding-behavior data, especially the addition of ECA_Conv, which provided the most significant increase in the mean values of the detection box and mask average precision among all the single improvement strategies because ECA_Conv can expand the sensing field while reducing the loss of practical features and combining the global contextual information to extract detailed features of the cow’s head and feed pile, proving the effectiveness of the method. For the complex and changing group feeding environment, the experimental results demonstrated that combining the three improved strategies can solve the problem of overlapping and interfering cow heads to a certain extent.

### 4.4. Effectiveness and Analysis of Picking Behavior Recognition

To demonstrate the improvement effect of the RefineMask-CEG model more intuitively, three models, namely, the classical model MaskRCNN, the original model RefineMask, and the improved model RefineMask-CEG, were selected to identify and segment the feeding-behaviors of dairy cows according to various scenarios, such as lighting conditions, shooting angle, and feeding background, respectively.

**Figure 8:**
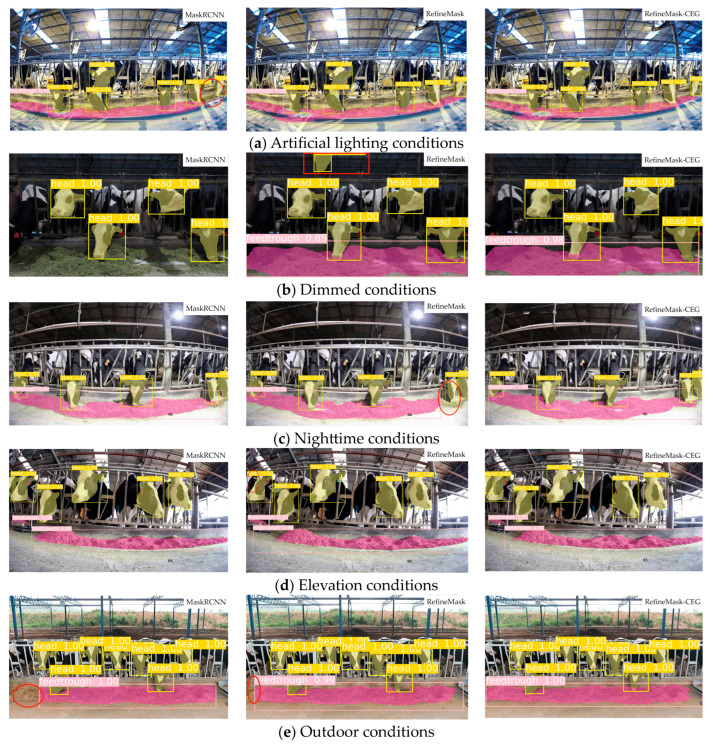
Visualization of the three models.

Figure 8a–c represent the visualizations of cow feeding-behavior under artificial lighting conditions, dim environments, and at night, respectively. As can be seen from the red box in the figure, the MaskRCNN model, under artificial lighting conditions, has leakage detection for the right edge of the feed pile. In the dark environment, the MaskRCNN model has a severe leakage detection problem for the feed pile, and the RefineMask model has a wrong-detection problem for the cow’s head. In a nighttime environment, the RefineMask model has leakage detection for the right edge of the feed pile. However, the improved RefineMask-CEG model performs well under all lighting conditions. This partly explains that the existing model for the feed pile segmentation effect is strongly affected by the lighting conditions, and the dimmer the environment, the poorer the model’s performance ability. Figure 8d shows the visualization effect of cow feeding-behavior under elevation conditions, and there is no noticeable difference among the three models, indicating that the model recognition is less affected by the angle. Figure 8e shows the recognition segmentation effect of different models under an open-air environment. It is evident that MaskRCNN to RefineMask has incomplete segmentation of the left side of the heap detection. Still, the segmentation of the feed pile of the RefineMask-CEG model is complete under the same picture, and the recognition segmentation effect of the improved model is significantly improved.

### 4.5. Comparison of Feeding-Time Calculations for Dairy Cows

In order to further validate the accuracy of the RefineMask-CEG model in identifying cow feeding-behavior and calculating cow feeding time in group farming environments, we conducted a comparative analysis of MaskRCNN, RefineMask, and RefineMask-CEG. For this purpose, we used a 120 s video of a multi-target cow-feeding to compare the real cow feeding time with the model-calculated cow feeding time. Table 6 comprehensively summarizes the results of the comparison.

The error between the algorithm and the manual is calculated according to the average absolute error formula, as shown in Equation (13):

(13) $$MAE=\frac{1}{n}\sum_{i=1}^{n}\left|{\hat{y}}_{i}-y_{i}\right|$$

where n represents the number of samples. In addition, ${\hat{y}}_{i}$ represents the predicted value and $y_{i}$ represents the true value.

Using the data in the table, the absolute errors of MaskRCNN, RefineMask, and RefineMask-CEG were calculated, respectively, and we obtained 14.62, 2.62, and 1.78. From the data, we can see that the RefineMask-CEG model has a low error value, and that it can achieve a more accurate recognition of the feeding-behavior of cows in the peak period of the cows’ feeding.

## 5. Conclusions

Based on the results of ablation tests performed by the heap-improved RefineMask-CEG model, as well as comparative tests with other example segmentation models and visualization analysis, the following conclusions can be drawn: In the ablation test, the number of parameters of the improved RefineMask-CEG instance-segmentation model is 49.96 M. The mean average precision of the detection box and the mean average precision of the mask are both 96%, which are 1.2% and 0.8% higher than those of the MaskRCNN model and 0.7% higher than that of the original RefineMask model, indicating that the improved model has a higher recognition-segmentation precision without a corresponding surge in the number of parameters, while maintaining a higher recognition segmentation precision. The improved RefineMask-CEG model outperforms the RefineMask model on the cow feeding-behavior dataset in comparative tests and visual analyses and also exceeds the mainstream instance-segmentation model. The enhanced model has higher precision in recognizing the head of the cow, and there is no leakage and misdetection phenomenon for the cow’s head. At the same time, based on the model’s accurate segmentation results of the cow’s head and the feed pile, the recognition of the cow’s feeding-behavior during the peak feeding period was achieved, which provides practical technical support for exploring and analyzing the relationship between the cow’s feeding-behavior and the amount of food intake under the group feeding mode.

In this paper, a series of exploratory researches on the non-contact recognition method of cow feeding-behavior were carried out by applying vision technology and deep learning technology to achieve the accurate segmentation of cow head and feed pile, as well as the recognition of cow feeding-behavior during the peak feeding period, findings which can be further developed, based on this work, in the future. In addition, current research is in the technology-discovery phase. The current study addresses the peak feeding period, and it is believed that cows in a hungry state will start feeding when they come into contact with the feed pile. However, outside of the peak feeding period, cows may sniff and arch during feeding, in which case the cow’s mouth will come into contact with the feed pile, but it will not feed. Therefore, in the future, we will focus on breaking the existing constraints and using action-recognition algorithms to exclude cases where cows touch the feed pile but do not feed, in order to further improve the accuracy and generalizability of identifying cows’ feeding-behavior.

## Figures and Tables

**Table 1:** Definition of labels for the dairy cow feeding behavior dataset.

Label Category	Label Description	Label Name	Number
Cow’s head	Cow head area outside the restriction fence	head	4291
Feeding trough	Banded irregular feed pile below limit fence	feed trough	1581

**Table 2:** Dairy cow feeding-behavior dataset.

	Number of Videos	Number of Images	Image Enhancement Methods	Image Resolution
Training set	192	800	Mosaic enhancement	3840 pixels × 100 pixels
Validation set	24	100	Mosaic enhancement	3840 pixels × 100 pixels
Test set	24	100	Mosaic enhancement	3840 pixels × 100 pixels
Total	240	1000	Mosaic enhancement	3840 pixels × 100 pixels

**Table 3:** Model experiment results.

Model	Detection Box Average Precision Mean/%	Mask Average Precision Mean/%	The Size of the Model Parameter/M	Frames per Second/FPS
MaskRCNN	97.1	97.5	44.17	2.3
MSRCNN	97.8	97.4	60.51	2.4
Point_Rend	97.1	97.6	59.99	1.9
Cascade_MaskRCNN	96.9	97.4	77.10	1.6
ConvNet_V2	97.7	97.3	110.59	2.7
RefineMask	97.6	97.6	48.34	1.4
RefineMask-CEG	98.3	98.3	49.96	0.8

**Table 4:** Comparison of average precision of target detection and segmentation in different categories.

	Head		Feed Trough	
Model	Average Precision of Detection Box/%	Average Precision of Mask/%	Average Precision of Detection Box/%	Average Precision of Mask/%
MaskRCNN	96.4	96.4	97.9	98.7
MSRCNN	96.4	96.2	99.2	98.6
Point_Rend	96.4	96.4	97.8	98.8
Cascade_MaskRCNN	96.4	96.4	97.4	98.4
ConvNetv2	96.5	96.2	98.9	98.4
RefineMask	96.5	96.5	98.7	98.7
RefineMask-CEG	97.3	97.3	99.3	99.2

**Table 5:** Results of ablation experiments.

Model	Accuracy Precision of Cow Head Detection Box/%	Average Precision of Feed Pile Detection Box/%	Mean Average Precision of Detection Box/%	Accuracy Precision of Cow Head Mask/%	Average Precision of Feed Pile Mask/%	Mean Average Precision of Mask/%
RefineMask	96.5	98.7	97.6	96.5	98.7	97.6
RefineMask + CB_Conv	97.3	98.9	98.1	96.4	99.0	97.7
RefineMask + ECA_Conv	97.3	99.3	98.3	96.4	99.3	97.9
RefineMask + CB + ECA	97.4	98.9	98.2	97.4	98.7	98.1
RefineMask-CEG	97.3	99.3	98.3	97.3	99.2	98.3

RefineMask + CB_Conv denotes the RefineMask network after adding the CB_Conv structure to the Refinemask’s original network. RefineMask + ECA_Conv represents the RefineMask network after adding the ECA_Conv structure to the Refinemask’s original network. RefineMask + CB + ECA denotes the RefineMask network after adding both CB_Conv and ECA_Conv structures to the actual network of Refinemask. RefineMask-CEG: signifies the simultaneous incorporation of CB_Conv, ECA_Conv, and the GIoU loss function into the RefineMask network.

**Table 6:** Calculation of feeding times for dairy cows.

Dairy Cow Number	Actual Value of Cow Feeding Time/s	Calculated Value of Cow Feeding-Time/s		
		MaskRCNN	RefineMask	RefineMask-CEG
Cow1	41	34.3	37.8	43.3
Cow2	56	35.2	53.1	58.2
Cow3	56	32.7	56.8	56.0
Cow4	52	30.8	46.6	53.7
Cow5	19	17.9	19.8	21.7

## Data Availability

The original contributions presented in the study are included in the article, further inquiries can be directed to the corresponding author/s.

## References

[1] Mulligan F., O’grady L., Rice D., Doherty M. (2006). A herd health approach to dairy cow nutrition and production diseases of the transition cow. Anim. Reprod. Sci..

[2] Unold O., Nikodem M., Piasecki M., Szyc K., Maciejewski H., Bawiec M., Dobrowolski P., Zdunek M. (2020). IoT-based cow health monitoring system. International Conference on Computational Science.

[3] Améndola L., Solorio F.J., Ku-Vera J.C., Améndola-Massioti R.D., Zarza H., Mancera K.F., Galindo F. (2019). A pilot study on the foraging behaviour of heifers in intensive silvopastoral and monoculture systems in the tropics. Animal.

[4] Sahu B.K., Parganiha A., Pati A.K. (2020). Behavior and foraging ecology of cattle: A review. J. Vet. Behav..

[5] Lima J.A.d.C., Fernandes H.J., da Silva A.G., Franco G.L., Rosa E.P., Falcão Y.d.S., Paiva L.M. (2020). Ingestive diurnal behaviour of grazing beef cattle. Semin. Ciências Agrárias.

[6] Mee J.F., Boyle L.A. (2020). Assessing whether dairy cow welfare is “better” in pasture-based than in confinement-based management systems. N. Z. Vet. J..

[7] He K., Gkioxari G., Dollár P., Girshick R. Mask r-cnn. Proceedings of the IEEE International Conference on Computer Vision.

[8] Martins L.F., Wasson D.E., Hristov A.N. (2022). Feeding dairy cows for improved metabolism and health. Anim. Front..

[9] Qiao Y., Guo Y., Yu K., He D. (2022). C3D-ConvLSTM based cow behaviour classification using video data for precision livestock farming. Comput. Electron. Agric..

[10] Magan J.B., O′Callaghan T.F., Kelly A.L., McCarthy N.A. (2021). Compositional and functional properties of milk and dairy products derived from cows fed pasture or concentrate-based diets. Compr. Rev. Food Sci. Food Saf..

[11] Williams L.R., Moore S.T., Bishop-Hurley G.J., Swain D.L. (2020). A sensor-based solution to monitor grazing cattle drinking behaviour and water intake. Comput. Electron. Agric..

[12] Gupta H., Jindal P., Verma O.P., Arya R.K., Ateya A.A., Soliman N.F., Mohan V. (2022). Computer vision-based approach for automatic detection of dairy cow breed. Electronics.

[13] Achour B., Belkadi M., Saddaoui R., Filali I., Aoudjit R., Laghrouche M. (2022). High-accuracy and energy-efficient wearable device for dairy cows’ localization and activity detection using low-cost IMU/RFID sensors. Microsyst. Technol..

[14] Bloch V., Pastell M. (2020). Monitoring of cow location in a barn by an open-source, low-cost, low-energy bluetooth tag system. Sensors.

[15] Aoughlis S., Saddaoui R., Achour B., Laghrouche M. (2021). Dairy cows’ localisation and feeding behaviour monitoring using a combination of IMU and RFID network. Int. J. Sens. Netw..

[16] Meunier B., Pradel P., Sloth K.H., Cirié C., Delval E., Mialon M.M., Veissier I. (2018). Image analysis to refine measurements of dairy cow behaviour from a real-time location system. Biosyst. Eng..

[17] Mancuso D., Castagnolo G., Porto S.M.C. (2023). Cow Behavioural Activities in Extensive Farms: Challenges of Adopting Automatic Monitoring Systems. Sensors.

[18] Mancuso D., Castagnolo G., Parlato M.C., Valenti F., Porto S.M. (2023). Low-power networks and GIS analyses for monitoring the site use of grazing cattle. Comput. Electron. Agric..

[19] Adrion F., Keller M., Bozzolini G.B., Umstatter C. (2020). Setup, test and validation of a UHF RFID system for monitoring feeding behaviour of dairy cows. Sensors.

[20] Stygar A.H., Gómez Y., Berteselli G.V., Dalla Costa E., Canali E., Niemi J.K., Llonch P., Pastell M. (2021). A systematic review on commercially available and validated sensor technologies for welfare assessment of dairy cattle. Front. Vet. Sci..

[21] Chelotti J.O., Vanrell S.R., Milone D.H., Utsumi S.A., Galli J.R., Rufiner H.L., Giovanini L.L. (2016). A real-time algorithm for acoustic monitoring of ingestive behavior of grazing cattle. Comput. Electron. Agric..

[22] Zambelis A., Wolfe T., Vasseur E. (2019). Validation of an ear-tag accelerometer to identify feeding and activity behaviors of tiestall-housed dairy cattle. J. Dairy Sci..

[23] Fuentes A., Yoon S., Park J., Park D.S. (2020). Deep learning-based hierarchical cattle behavior recognition with spatio-temporal information. Comput. Electron. Agric..

[24] Buijs S., Weller J., Budan A. (2023). When the measurement affects the object–Impact of a multi-part head/neck mounted wearable device on dairy cow behaviour, health and productivity. Appl. Anim. Behav. Sci..

[25] Achour B., Belkadi M., Filali I., Laghrouche M., Lahdir M. (2020). Image analysis for individual identification and feeding behaviour monitoring of dairy cows based on Convolutional Neural Networks (CNN). Biosyst. Eng..

[26] Kuan C.Y., Tsai Y.C., Hsu J.T., Ding S.T., Te Lin T. An imaging system based on deep learning for monitoring the feeding behavior of dairy cows. Proceedings of the 2019 ASABE Annual International Meeting, American Society of Agricultural and Biological Engineers.

[27] Porto S.M., Arcidiacono C., Anguzza U., Cascone G. (2015). The automatic detection of dairy cow feeding and standing behaviours in free-stall barns by a computer vision-based system. Biosyst. Eng..

[28] Yu Z., Liu Y., Yu S., Wang R., Song Z., Yan Y., Li F., Wang Z., Tian F. (2022). Automatic detection method of dairy cow feeding behaviour based on YOLO improved model and edge computing. Sensors.

[29] Bai Q., Gao R., Zhao C., Li Q., Wang R., Li S. (2022). Multi-scale behavior recognition method for dairy cows based on improved YOLOV5s network. Trans. Chin. Soc. Agric. Eng..

[30] Lassen J., Thomasen J.R., Borchersen S. (2023). Repeatabilities of individual measures of feed intake and body weight on in-house commercial dairy cattle using a 3-dimensional camera system. J. Dairy Sci..

[31] Bloch V., Frondelius L., Arcidiacono C., Mancino M., Pastell M. (2023). Development and Analysis of a CNN-and Transfer-Learning-Based Classification Model for Automated Dairy Cow Feeding Behavior Recognition from Accelerometer Data. Sensors.

[32] Pavkin D.Y., Nikitin E.A., Shilin D.V., Belyakov M.V., Golyshkov I.A., Mikhailichenko S., Chepurina E. (2023). Development Results of a Cross-Platform Positioning System for a Robotics Feed System at a Dairy Cattle Complex. Agriculture.

[33] Bello R.W., Mohamed A.S.A., Talib A.Z. (2021). Contour extraction of individual cattle from an image using enhanced Mask R-CNN instance segmentation method. IEEE Access.

[34] Zhang G., Lu X., Tan J., Li J., Zhang Z., Li Q., Hu X. Refinemask: Towards high-quality instance segmentation with fine-grained features. Proceedings of the IEEE/CVF Conference on Computer Vision and Pattern Recognition.

